# *When*, *How*, and *If* Intelligence Testing Matters for Academic Achievement

**DOI:** 10.3390/jintelligence14100262

**Published:** 2026-10-16

**Authors:** Meredith C. Frey, Christopher R. Niileksela

**Affiliations:** 1 Department of Psychology, Otterbein University, Westerville, OH 43081, USA; 2 Department of Educational Psychology, University of Kansas, Lawrence, KS 66045, USA; chrisn@ku.edu

## 1. Introduction

Deary et al. once called intelligence testing “…one of the most widespread yet most controversial exports from academic psychology to the real world…” (Deary et al., 2007, p. 13). In the same study, these researchers found a correlation of 0.81 between a latent *g* factor extracted from a cognitive battery administered at age 11 and later general educational attainment of 16-year-old students. Nearly a decade later, a meta-analysis comprising 240 studies and over 100,000 participants by Roth and colleagues found an average correlation of 0.54 between intelligence and school grades. They concluded that “…intelligence has substantial influence on school grades and thus can be regarded as one of the most (if not the most) influential variables in this context” (Roth et al., 2015, p. 126). It is hard to imagine stronger evidence for the general relationship between intelligence and academic achievement. Yet, the use of intelligence testing as a way to help understand academic achievement and make educational decisions has faced continued scrutiny. 

The controversy around using intelligence tests has continued, primarily outside of psychology, and has included calls for the abandonment of these tools (e.g., Tough, 2020; Rim, 2021; Headlee, 2013). The reasons for this controversy are multifaceted, including difficulties defining intelligence, which is “an elusive construct for an elusive field” (Sternberg & Detterman, 1986, p. viii), if there is such thing as “general intelligence” or if it is a statistical artifact (e.g., Horn & Blankson, 2012; Kovacs & Conway, 2016), what the scores on intelligence tests represent (e.g., Beaujean & Benson, 2019; Beaujean et al., 2018), and the consequences of decisions made about people that are based in part or solely on the use of those scores (van Hoogdalem & Bosman, 2024). In educational settings, much of the controversy seems to involve the validity of intelligence test scores, whether they may be helpful for understanding learning (including learning assets and learning difficulties), and what that understanding may mean for a person’s education, especially regarding decisions made on the basis of intelligence test scores. 

In this Editorial, we argue that intelligence testing continues to demonstrate practical utility, with the caveat that the use of intelligence test scores is nuanced and requires careful thought and evidence-based reasoning when these scores are used for educational purposes. We focus on three domains of academic life in this Special Issue: (1) understanding cross-sectional changes, means, and relationships between intelligence and academic skills, (2) identifying giftedness/talent, and (3) understanding learning and intellectual disabilities.

The research reported in this Special Issue contains samples drawn from across the ability range—from mild nonspecific intellectual disability to giftedness—and from several countries. The collected articles reflect advances in understanding post-pandemic “learning loss” across the ability spectrum in the U.S., a more nuanced understanding of the development of reading and writing ability through middle childhood and adolescence among students with intellectual impairment, a novel way of identifying domain-specific giftedness based on a common measure of fluid reasoning ability, a framework for establishing the utility of intelligence tests for understanding achievement, and a meta-analysis of the relationships between general, broad, and narrow cognitive abilities with mathematics skills. The common theme across these papers is that an understanding of cognitive abilities, both general and specific, can provide useful information in understanding learning difficulties and assets and may ultimately assist when determining appropriate educational strategies and interventions for at-risk students. 

This Editorial is organized as follows: First, we provide a brief review of the articles included in this Special Issue and discuss the implications of those studies. Second, we review several open questions that provide a foundation for future research and inquiry that arose from our review of these articles. Finally, we end with some general conclusions about the use of intelligence tests and the relationship between intelligence and academic achievement.

## 2. Summary of Special Issue Studies

### 2.1. Understanding Cross-Sectional Changes

Much research has been devoted to understanding secular changes in IQ across decades and generations since the 1980s (see Flynn, 2020, for a comprehensive review). More recently, researchers have been investigating age-related differences in the relationship between cognitive ability and academic achievement, suggesting a mutualism model wherein cognitive abilities facilitate the development of academic skills, which in turn facilitates the development of cognitive abilities (cf. Peng et al., 2018, 2019). However, these changes in relationships across age groups, when present, appear to be rather smaller than the secular IQ gains. In this Special Issue, Helal and Wai (2026) examined the changes in cognitive ability and academic achievement in a U.S. sample of more than 10,000 U.S. students, both before and after the COVID-19 pandemic. Their results indicate that, while ability gaps did not increase (indeed, they appeared to decrease across certain demographic characteristics from pre-pandemic levels) compared to the relationship between cognitive ability and achievement pre-pandemic, the relationship between cognitive ability and achievement weakened post-pandemic. This was especially pronounced at lower levels of cognitive ability. Findings like these suggest that high-ability students are able to acquire academic skills well in a range of environments, whereas lower-ability students may have more difficulty acquiring academic skills when educational systems are disrupted. Educators should be mindful of the instruction and practice needs of lower-ability students, particularly around major events that disrupt access to education.

### 2.2. Giftedness Identification

Intelligence tests have been used to identify giftedness for decades, and they are effective at differentiating among gifted individuals even at the highest levels of ability (see Lubinski & Benbow, 2021). Their use has been scrutinized, and alternative methods that are intended to reduce disparities among majority and minority populations have been proposed. Evidence suggests that these alternative methods measure something different from traditional intelligence tests, and, while they may narrow the difference between majority and minority populations, they do not necessarily account for the disparities among these groups (Acar et al., 2016; Hodges et al., 2018). The identification of students who qualify for gifted services in the U.S. is complex and varies substantially across states and school districts (Greene et al., 2025). According to some, the use of a single test score is not recommended for gifted identification (Worrell, 2009), although standardized intelligence and academic achievement test scores have been some of the most prominent and consistent measures used to identify students with high cognitive and academic skills (Delgado-Valencia et al., 2025). In this Special Issue, Peperkorn and Wegner (2026) adapted the well-established matrix reasoning task—historically an excellent measure of fluid reasoning (G*f*)—to measure areas of specific giftedness in biological sciences. This innovative approach has the same format of matrix reasoning tasks, but stimuli include content related to biological organisms and processes. Notably, their matrix reasoning task contains a substantial range of item difficulty, it does not manifest significant differential item functioning across genders or grade levels, and their results showed that students who were considered gifted in biology performed better than a comparison group on this task. Their research shows that these types of tasks may be useful for identifying specific areas of giftedness beyond broad academic and cognitive ability test scores.

### 2.3. Intellectual and Specific Learning Disabilities

The use of intelligence tests in the identification of learning difficulties, especially specific learning disabilities, has been controversial, especially in the field of school psychology (Fletcher & Miciak, 2017; Schneider & Kaufman, 2017). Whereas the use of intelligence tests for identifying *intellectual disability* has been well-established and current practices are accepted by the field (Fletcher & Miciak, 2024), their use in identifying *specific learning disabilities* has been less clear. No answer or approach has been proposed and accepted by the field as a “gold standard” method for identifying learning disabilities. Intelligence tests were an essential part of the process identifying learning disabilities in the past when a learning disability was defined as having a “significant” gap between an IQ score and their academic achievement. This definition was based on the idea that this gap represents “unexpected underachievement”, and although the approach is a clear operationalization of this concept, the problems with this approach as a single method for identifying learning disabilities were significant (Stuebing et al., 2002). Researchers have proposed other approaches, some that do not rely on comprehensive intelligence tests (e.g., response to intervention, hybrid model from Fletcher et al., 2019) and others that do rely on intelligence tests, although the focus has primarily been on identifying specific cognitive abilities (e.g., working memory, fluid reasoning) that may partially explain why a student is having difficulty learning. Although an argument can be made that focusing only on the difficulty with learning is important (e.g., if someone has a reading difficulty, focus on services to remediate that difficulty), some argue that the incorporation of scores from intelligence tests can help provide a better understanding of the student and their individual needs (Peng, 2025).

In this Special Issue, Niileksela et al. (2026) integrated existing datasets from norm-referenced tests in a meta-analytic investigation of the relationships between cognitive abilities and mathematics skills. In an analysis of more than 500 correlation matrices, the authors found empirical support for a hierarchical acquisition of mathematics skills, where Stratum II factors of cognitive ability explained differences in mathematics skills. When considering *for whom* and *under what circumstances* intelligence testing may be beneficial in the context of children’s education, the study offers compelling evidence of a particular utility: educators and practitioners can use the relationships among G*f*, G*c*, and mathematics skills to clarify if low mathematics achievement is the result of reasoning difficulty (G*f*) or content knowledge deficiency/instructional gap (G*c*). As these antecedents suggest different remedies, interventions can be tailored to more effectively address the nature of the deficit. 

Cognitive ability testing, especially obtaining an estimate of general intelligence, is an essential component of an intellectual disability (ID) diagnosis. However, beyond general ability deficits, a more refined assessment has the potential to foster intervention and placement decisions that may maximally serve people with ID. In this Special Issue, Sajewicz-Radtke et al. (2025) used a fuzzy cluster analysis to identify areas of relative weakness in cognitive–linguistic competencies among a large sample of children and adolescents with mild nonspecific intellectual disability. Their results suggest a fine-grained ability analysis also helps identify areas of specific deficit and informs intervention strategies among youth with intellectual disabilities. The use of fuzzy cluster analysis is a unique approach because of openly accepting the inherent error that exists in test scores, and, rather than focusing on placing people in specific groups, the approach provides probabilities of group membership, explicitly modeling the uncertainty of group membership.

### 2.4. Dynamic Testing and Learning

Büchli et al. (2026) addressed dynamic assessment, which could be used as a method for measuring learning, a concept that is relevant for education. Their study focused on dynamic assessment using a fluid reasoning test (figural analogies), where participants were either provided with no feedback, digitally mediated feedback (no person was involved in providing feedback), or socially mediated feedback (a person was involved in providing feedback). The goals were to determine if providing feedback improves post-test performance and if the type of feedback matters. Providing feedback on performance and measuring how a student learns and improves in their task performance were hypothesized to provide an estimate of learning potential. They found that those who received feedback performed better than those in the control group, which is what would be expected for those receiving feedback on their performance. Additionally, those receiving socially mediated feedback performed better than those receiving digital feedback. Although they did not find that academic performance was better predicted by those who received feedback, the study provides an initial examination of how providing feedback on tasks can improve performance on cognitive ability tests, even the results on fluid reasoning tasks, which may seem to be challenging to change because they are related to cognitive abilities or processes that are viewed as less pliable.

### 2.5. Setting a Research Agenda

Pedraja-Rejas et al. (2026) propose a structured method of answering the central questions of this Special Issue: for whom and under what circumstances intelligence testing is useful for understanding academic achievement. The current state of intelligence testing in academic achievement is described by Pedraja-Rejas et al. as a paradox wherein “…cognitive measures continue to show meaningful predictive relationships with educational outcomes, yet their legitimacy and practical acceptability in education decision-making have weakened” (p. 2). Yet, rather than ceding the field, Pedraja-Rejas et al. constructed a decision utility framework, where they propose focusing on intelligence testing where the framework makes the most impact. Intelligence tests likely add meaningful information at transitions to novel environments (e.g., a shift from secondary school to university or from traditional to advanced tracks), in cases where prior opportunity to appropriate instructional materials/methods was limited, in targeted applications rather than blanket testing, or to aid in policy decisions. Perhaps the greatest strength of the authors’ approach is explaining, in real terms and with implementation checklists, how to use the information gained from intelligence testing to make better decisions, evaluate error costs, and empirically justify intelligence testing in specific contexts.

## 3. Open Questions

Overall, the articles in this Special Issue address a variety of topics about the use of intelligence testing as relates to academic achievement. These articles also suggest several important avenues for future research that may be helpful for continuing to understand how cognitive abilities and academic achievement are related. These open questions are reviewed below, with commentary about how they may be addressed and investigated through further inquiry. 

### 3.1. What Methodological or Analytical Advances Will Increase Our Understanding of the Relationships Between Intelligence Testing and Academic Achievement?

#### 3.1.1. Focusing on Moderating Variables

Research on cognitive abilities and academic achievement has been largely driven by examining individual differences, with a focus on correlational methods (e.g., regression, exploratory and confirmatory factor analysis, and structural equation modeling). Whereas issues of moderation have been examined through these paradigms (e.g., studies on measurement invariance), a more nuanced program of research that focuses more on the different “when and for whom” relationships among cognitive abilities and academic achievement is open for more examination. This includes focusing on the differences in how cognitive and academic variables are related across the distribution of test scores. For example, does the relationship between cognitive abilities and academic outcomes differ among people with lower or higher levels of cognitive ability? Is there a functional form that describes how that relationship changes across the distribution? Spearman’s Law of Diminishing Returns (SLODR) is one finding that is important here, where the correlations among tests are stronger for those with lower general ability than those with higher general ability. However, these relationships and differences could be much more nuanced than previously examined. For example, Hajovsky et al. (2025b) used moderated nonlinear factor analysis to examine whether the relationships between cognitive abilities and reading skills differed by general intellectual ability and age. They found several significant differences, such as the strength of the relationship between working memory and basic reading skills increasing with age but only for those with lower levels of general cognitive ability, whereas the opposite pattern was present for those with higher levels of general cognitive ability. The authors of several of the studies in this Special Issue examined some of these important contextual differences using new assessment methods and approaches (e.g., dynamic assessment and using context-specific items to test reasoning) and by focusing on quantile regression to examine the differences in the relationships across the ability distribution. We should not assume that all group-level research results can be generalized to the individual (this may be extremely rare, see Molenaar, 2004), and understanding these relationships in different parts of the cognitive and academic ability distribution would be a helpful extension of knowledge. 

Helal and Wai (2026) highlight the importance of examining the differences in the relationships among variables across different levels of independent and dependent variables using quantile regression. When we estimate a correlation, the assumption is that the correlation coefficient equally applies to all parts of a distribution, but methods such as quantile regression can help show how those relationships may not hold across a distribution of scores. If correlations are stronger for people in different parts of a distribution, the implication is that some cognitive variables have a stronger or weaker influence on academic variables for different people. What needs to be examined further is the extent of these differences across different types of variables and to what extent those differences have practical implications for understanding learning differences. Some researchers have found that these relationships change. For example, Hajovsky et al. (2020) found that verbal comprehension was a strong predictor of reading comprehension, but this relationship was stronger for early elementary students with lower levels of reading comprehension. This implies that verbal comprehension can better differentiate good and poor comprehenders for those with lower levels of reading comprehension compared to those with higher levels of reading comprehension. 

#### 3.1.2. Understanding the Utility of Dynamic Testing

Research on dynamic assessment has not been as common in recent years, especially regarding cognitive ability testing (see Grigorenko, 2009, for an overview of the history of dynamic assessment and its parallels to more recent responses to intervention models of service delivery). Dynamic assessment can be difficult to research because of being challenging to standardize, especially in relation to providing feedback and teaching. Although some evidence suggests dynamic testing is predictive of outcomes, less evidence exists for its consequential validity (Tiekstra et al., 2016). However, dynamic assessment can be a useful process that mirrors important aspects of education: understanding what students can do, what they need to learn, teaching them those skills, and then evaluating whether they have attained an adequate level of competence. What remains to be seen is whether evaluating learning through these tasks is beneficial. For example, does knowing if a student learns new skills with more or less support provide added value when predicting academic outcome, whether those outcomes are grades, test scores, or other important milestones (e.g., graduating high school, enrolling in and completing college degrees)? If we find that a student has difficulty learning new skills in a dynamic assessment, can that help teachers better match that student with more appropriate instructional methods? Whereas rate of learning has been used for some aspects of assessment (e.g., curriculum-based assessment and response to intervention, using rate of improvement in reading or mathematics skills to determine if someone should or should not receive special education services), this has not been extensively studied with nonacademic tests (e.g., reading, mathematics, or writing). Some dynamic assessments with more standardized procedures have recently been developed (e.g., the Dynamic Measures of Narrative Language and Decoding [DYMOND], Petersen et al., 2025), but further research is needed to better understand how the learning part of the assessment might be used to better understand learning difficulties and assets and whether the assessment provides more information than the scores that are obtained in static assessments.

#### 3.1.3. Developing Context-Specific Test Stimuli

Peperkorn and Wegner (2026) show that creating matrix reasoning items that include domain-specific content in biology was advantageous to those who had specific content strengths in biology. In other words, this approach may be useful for helping to identify students who are able to apply reasoning skills to specific content areas. With continued technological developments, matrix reasoning tasks could be developed that include content from a wide variety of specific content areas. Their results suggest a new avenue for the development of domain-specific assessments.

### 3.2. Will Interventions Based on a More Precise Understanding of the Relationship Between Intelligence and Academic Achievement Improve Educational Outcomes?

#### Targeted Mathematics Intervention

The question of whether cognitive ability testing adds value to understanding how we might intervene with students continues to be discussed in the literature (e.g., Hajovsky et al., 2025a). Although cognitive ability data should not be used to drive intervention selection (e.g., Burns et al., 2016), whether certain cognitive ability measures can enhance the understanding of what children may need for intervention may be useful in specific cases (e.g., Fuchs et al., 2014). Peng and Goodrich (2020) argued that incorporating cognitive skills into intervention that are domain-specific may be a useful approach. For example, rather than focusing on training working memory as a separate skill using a task that is disconnected from real-world academic skills (e.g., training on the *N*-back task) where far transfer can be negligible (Melby-Lervåg et al., 2016), training on tasks that incorporate similar content or materials may assist with near-transfer effects. This ties into the study by Peperkorn and Wegner (2026), where the use of content-specific items within a reasoning task could be a useful approach for developing reasoning skills within a specific academic content area. This provides a unique field of study to determine whether these types of tests are useful for understanding and developing cognitive and academic skills. 

### 3.3. What Are the Consequences of Intelligence Testing?

#### 3.3.1. Thresholds, Access, and Opportunity

Intelligence tests are used in a variety of contexts, including formal academic institutions, workplaces, and the legal system. The consequences of intelligence testing are similarly varied (e.g., placement in enrichment or remediation, eligibility for social and governmental services, selection and accommodation, or decisions regarding capital punishment). All measurement contains error; as Schneider et al. (2026) demonstrated, even well-designed IQ tests only provide us with an estimate of a person’s IQ, and estimates resulting from repeated testing sometimes reside on opposite sides of a relevant threshold. Although the forensic application of IQ testing discussed in Schneider et al. is not the focus of this Special Issue, when we consider the role of intelligence testing in academic achievement, we must remain similarly mindful of the consequences of assessment. What support, enrichment, or academic opportunities are available to individuals on either side of a relevant threshold, and how certain are we that we have placed a student on the “right side of the line”? Furthermore, as Lubinski (2025) noted, efforts to reduce selectivity for some learning environments may harm talented students, and the outcomes extend far beyond the individuals being assessed. The consequences of mismatch can be disastrous because “we need both bridges and legal contracts that hold up when tested” (p. 2).

#### 3.3.2. Decision Utility

In a study in this Special Issue, Pedraja-Rejas et al. (2026) considered not only the incremental validity of cognitive ability testing but also the decision utility of such tests, which is particularly relevant in higher education achievement. Although researchers have long known that grades and tests together are the best predictor of postsecondary success and that common criticisms of tests (e.g., they are mostly measures of SES) are not empirically supported (e.g., Kuncel & Hezlett, 2010; Sackett et al., 2009), our understanding of success in college may be shifting.

For example, Harvard College’s released grade report demonstrates a significant increase in the assignment of A grades from 24% of the time 20 years ago to 60.2% of the time in 2025 (Claybaugh, 2025). College Board research indicates that college grade inflation is most common with selective private institutions (Westrick et al., 2024), yet the researchers also found that faculty are reporting students with lower levels of preparation (mirrored by lower SAT scores) and increased pressure to assign higher grades across institutions.

This is not to say that grade inflation is necessarily negative. However, it means that grade inflation alters how grades can be used to understand academic achievement. Inflated grades introduce a ceiling effect that can impact the utility of grades for predicting outcomes (e.g., underestimating regression parameters), which ultimately may negatively affect the reliability and validity of grades as measures of academic achievement (Uttl, 2005). For example, if grades become a measure of minimum competence (e.g., a class is passed or not passed) then the distribution of grades (and ultimately GPA) changes from a continuum that differentiates those who performed well from those who performed average and those who performed poorly to a dichotomy that differentiates people who met a minimum level of competence from those who did not. This type of differentiation may be appropriate for certain courses or programs where is, but increased differentiation may be necessary at other times. For example, some physics programs require a high level of mathematics knowledge and competence. When choosing students to join a rigorous and prestigious physics program, having increased differentiation of grades in previous mathematics courses can provide important information beyond the score on the Quantitative section of the GRE. Admitting applicants to programs that they may not be prepared to join because they do not have the necessary mathematics skills is not fair to those applicants, unless the program has supports in place to ensure that students are able to acquire the necessary mathematics skills. Accessibility is important, but accessibility without accommodation does not set up incoming students for success. The question then becomes: in the presence of grade inflation, are pre-admissions tests (which are highly correlated with measures of general cognitive ability) still useful for predicting achievement outcomes? 

General cognitive ability correlates with current and future academic achievement, but prior achievement also correlates with current and future academic achievement. Intelligence test scores must not just add to the prediction of success but also be likely to change decisions to be useful. More specifically, when resources are plentiful, seats are many, or probability of current or future achievement is already well-understood by prior achievement, then intelligence testing adds little to the decision framework. Pedraja-Rejas et al. (2026) argue that practitioners and policy makers should also consider the error cost associated with decisions based on cognitive ability assessment. If the costs of false positives (e.g., mismatch between a student and a program, especially if a student spends significant resources to join the program) or false negatives (e.g., underidentification of talented youth) are decreased by the application of intelligence testing, then including testing in the selection process could be beneficial.

#### 3.3.3. Resource-Constrained Environments

Much of the research on intelligence testing and post-secondary academic achievement has drawn from U.S. samples, though evidence from the U.K, continental Europe, Australia, and China also confirms the central finding that intelligence is important for tertiary school achievement. The evidence is ample that intelligence is well-measured and conforms to the same basic factor structure globally (Warne & Burningham, 2019; Pokropek et al., 2022). Yet, comparatively little is known about intelligence testing and tertiary school achievement in developing nations. A study on a small sample from Ethiopia showed that some cognitive variables, such as processing speed and working memory, were important predictors of grade point average (Kassaw & Demareva, 2025), which is a promising step, but more research outside of the developed world is needed.

#### 3.3.4. Beyond Tertiary Education

As a consequence of criterion changes in higher education achievement and the employment of a decision utility framework, the importance of intelligence testing may be shifting to selection for postgraduate education. Kuncel and Hezlett (2007) demonstrated in a review of published research that standardized admissions testing for graduate school (e.g., the GRE, LSAT, and MCAT) was significantly predictive of performance in graduate classes, faculty ratings, and more distal criteria such as licensing exams. We are not asserting that these exams are intelligence tests per se; however, they share characteristics with many tests of cognitive ability and merit consideration as we investigate the relationship between testing and academic achievement.

## 4. Conclusions

We opened this Special Issue to promote research elucidating *for whom* and/or *under what circumstances* intelligence testing plays a role in academic achievement, particularly regarding placement in educational programs (e.g., gifted programs, special education), post-secondary admissions and selection, and to help understand individual differences in academic achievement. The six papers contained herein provide a framework for deciding when to employ intelligence testing, several methodological/analytical advances in testing, and an increased understanding of the core cognitive components of achievement in specific domains.

Although the articles in this Special Issue help move the field forward in some specific areas, they also spur many more questions that must be considered. When we are deciding if we should administer an intelligence test for an academic purpose, we should be clear about why we are using it, if its use is appropriate, and if it will provide information that may improve decisions or understanding of a particular individual. Additionally, we must be mindful of the consequences, intended and unintended, of using those test scores that may have significant impacts on a particular individual. Ideally, these considerations should be made early and as part of the test development process (Caemmerer et al., 2026).

Generally, we find ourselves in a similar point as that referenced in Sternberg and Detterman’s (1986) symposium, a follow-up to a symposium in the *Journal of Educational Psychology* in 1921. We can report progress on understanding intelligence and the relationship between intelligence testing and achievement. Yet, we recognize the additional work required as our understanding of the uses, costs, benefits, and consequences of intelligence testing develops.

## References

[B1-jintelligence-14-00262] Acar S., Sen S., Cayirdag N. (2016). Consistency of the performance and nonperformance methods in gifted identification. Gifted Child Quarterly.

[B2-jintelligence-14-00262] Beaujean A. A., Benson N. F. (2019). The one and the many: Enduring legacies of spearman and thurstone on intelligence test score interpretation. Applied Measurement in Education.

[B3-jintelligence-14-00262] Beaujean A. A., Benson N. F., McGill R. J., Dombrowski S. C. (2018). A misuse of IQ scores: Using the dual discrepancy/consistency model for identifying specific learning disabilities. Journal of Intelligence.

[B4-jintelligence-14-00262] Burns M. K., Petersen-Brown S., Haegele K., Rodriguez M., Schmitt B., Cooper M., Clayton K., Hutcheson S., Conner C., Hosp J., VanDerHeyden A. M. (2016). Meta-analysis of academic interventions derived from neuropsychological data. School Psychology Quarterly.

[B5-jintelligence-14-00262] Büchli A., Beckmann J. F., Troche S. J. (2026). Differential effects of social and digital feedback interventions in the figural analogies learning test (FALT). Journal of Intelligence.

[B6-jintelligence-14-00262] Caemmerer J. M., Koslouski J. B., Briesch A., Chafouleas S. M., Melo B. (2026). Consequential validity-centered measure development: An illustration using the ESSY whole child screener. Journal of Psychoeducational Assessment.

[B7-jintelligence-14-00262] Claybaugh A. (2025). Re-centering academics at Harvard college: Update on grading and workload.

[B8-jintelligence-14-00262] Deary I. J., Strand S., Smith P., Fernandes C. (2007). Intelligence and educational achievement. Intelligence.

[B9-jintelligence-14-00262] Delgado-Valencia L., Delgado B., Navarro-Soria I., Torrecillas M., Rosales-Gómez M., Sánchez-Herrera M. d. l. C., Soto-Díaz M. (2025). The identification of giftedness in children: A systematic review. Education Sciences.

[B10-jintelligence-14-00262] Fletcher J. M., Lyon G. R., Fuchs L. S., Barnes M. A. (2019). Learning disabilities: From identification to intervention.

[B11-jintelligence-14-00262] Fletcher J. M., Miciak J. (2017). Comprehensive cognitive assessments are not necessary for the identification and treatment of learning disabilities. Archives of Clinical Neuropsychology.

[B12-jintelligence-14-00262] Fletcher J. M., Miciak J. (2024). Assessment of specific learning disabilities and intellectual disabilities. Assessment.

[B13-jintelligence-14-00262] Flynn J. R., Sternberg R. J. (2020). Secular changes in intelligence: The “Flynn effect”. The Cambridge handbook of intelligence.

[B14-jintelligence-14-00262] Fuchs L. S., Schumacher R. F., Sterba S. K., Long J., Namkung J., Malone A., Hamlett C. L., Jordan N. C., Gersten R., Siegler R. S., Changas P. (2014). Does working memory moderate the effects of fraction intervention? An aptitude–treatment interaction. Journal of Educational Psychology.

[B15-jintelligence-14-00262] Greene A. K., Dougé M. C. E., Maki K. E. (2025). State definitions, policies, and practices of gifted and talented identification: What difference does a decade make?. Gifted Child Quarterly.

[B16-jintelligence-14-00262] Grigorenko E. L. (2009). Dynamic assessment and response to intervention: Two sides of one coin. Journal of Learning Disabilities.

[B17-jintelligence-14-00262] Hajovsky D. B., Maki K. E., Niileksela C. R., McGill R. J. (2025a). Science and practice of identifying specific learning disabilities: Kind conversations about a wicked problem. Journal of Psychoeducational Assessment.

[B18-jintelligence-14-00262] Hajovsky D. B., Niileksela C. R., Robbins J., Sun Y. (2025b). Understanding contextual specificity in cognitive-reading relations: Moderation by age and IQ. Journal of Psychoeducational Assessment.

[B19-jintelligence-14-00262] Hajovsky D. B., Villeneuve E. F., Schneider W. J., Caemmerer J. M. (2020). An alternative approach to cognitive and achievement relations research: An introduction to quantile regression. Journal of Pediatric Neuropsychology.

[B20-jintelligence-14-00262] Headlee C. (2013). Is it time to get rid of IQ tests in schools? *[Radio broadcast transcript]*.

[B21-jintelligence-14-00262] Helal A. M., Wai J. (2026). Quantile regression of cognitive ability and achievement inequality before and after the pandemic in one state. Journal of Intelligence.

[B22-jintelligence-14-00262] Hodges J., Tay J., Maeda Y., Gentry M. (2018). A meta-analysis of gifted and talented identification practices. Gifted Child Quarterly.

[B23-jintelligence-14-00262] Horn J. L., Blankson A. N., Flanagan D. P., Harrison P. L. (2012). Foundations for better understanding of cognitive abilities. Contemporary intellectual assessment: Theories, tests, and issues.

[B24-jintelligence-14-00262] Kassaw C., Demareva V. (2025). Psychosocial and cognitive predictors of academic achievement among higher education students in Southern Ethiopia. PLoS ONE.

[B25-jintelligence-14-00262] Kovacs K., Conway A. R. A. (2016). Process overlap theory: A unified account of the general factor of intelligence. Psychological Inquiry.

[B26-jintelligence-14-00262] Kuncel N. R., Hezlett S. A. (2007). Standardized tests predict graduate students’ success. Science.

[B27-jintelligence-14-00262] Kuncel N. R., Hezlett S. A. (2010). Fact and fiction in cognitive ability testing for admissions and hiring decisions. Current Directions in Psychological Science.

[B28-jintelligence-14-00262] Lubinski D. (2025). Education, intelligence, placement, and selection: A discussion of paradoxes and fairness. Intelligence.

[B29-jintelligence-14-00262] Lubinski D., Benbow C. (2021). Intellectual precocity: What have we learned since Terman?. Gifted Child Quarterly.

[B30-jintelligence-14-00262] Melby-Lervåg M., Redick T. S., Hulme C. (2016). Working memory training does not improve performance on measures of intelligence or other measures of “far transfer”: Evidence from a meta-analytic review. Perspectives on Psychological Science.

[B31-jintelligence-14-00262] Molenaar P. C. M. (2004). A manifesto on psychology as idiographic science: Bringing the person back into scientific psychology, this time forever. Measurement: Interdisciplinary Research and Perspectives.

[B32-jintelligence-14-00262] Niileksela C. R., Hajovsky D. B., Cocar-Montenegro F. P., Schneider W. J., Flanagan D. P., Alfonso V. C. (2026). Cognitive-mathematics relations: A meta-analysis of norm-referenced standardized test batteries. Journal of Intelligence.

[B33-jintelligence-14-00262] Pedraja-Rejas L., Araneda-Guirriman C., Rodríguez-Ponce E. (2026). From incremental validity to decision utility: A framework for intelligence testing in education. Journal of Intelligence.

[B34-jintelligence-14-00262] Peng P. (2025). Understanding the relations between cognition and academic skills in the context of instruction. Advances in Child Development and Behavior.

[B35-jintelligence-14-00262] Peng P., Barnes M., Wang C., Wang W., Li S., Swanson H. L., Dardick W., Tao S. (2018). A meta-analysis on the relation between reading and working memory. Psychological Bulletin.

[B36-jintelligence-14-00262] Peng P., Goodrich J. M. (2020). The cognitive element model of reading instruction. Reading Research Quarterly.

[B37-jintelligence-14-00262] Peng P., Wang T., Wang C., Lin X. (2019). A meta-analysis on the relation between fluid intelligence and reading/mathematics: Effects of tasks, age, and social economics status. Psychological Bulletin.

[B38-jintelligence-14-00262] Peperkorn C., Wegner C. (2026). From abstract to domain-specific: Development and validation of matrix reasoning tasks for students in biology. Journal of Intelligence.

[B39-jintelligence-14-00262] Petersen D. B., Spencer T. D., Konishi-Therkildsen A. (2025). Dynamic measures of narrative language and decoding (DYMOND).

[B40-jintelligence-14-00262] Pokropek A., Marks G. N., Borgonovi F. (2022). How much do students’ scores in PISA reflect general intelligence and how much do they reflect specific abilities?. Journal of Educational Psychology.

[B41-jintelligence-14-00262] Rim C. (2021). It’s time to abolish the SAT *[Opinion]*.

[B42-jintelligence-14-00262] Roth B., Becker N., Romeyke S., Schäfer S., Domnick F., Spinath F. M. (2015). Intelligence and school grades: A meta-analysis. Intelligence.

[B43-jintelligence-14-00262] Sackett P. R., Kuncle N. R., Arneson J. J., Cooper S. R., Waters S. D. (2009). Does socioeconomic status explain the relationship between admissions tests and post-secondary academic performance?. Psychological Bulletin.

[B44-jintelligence-14-00262] Sajewicz-Radtke U., Łada-Maśko A. B., Jurek P., Olech M., Radtke B. M. (2025). Reading and writing abilities in students with mild nonspecific intellectual disability: A multivariate examination of literacy and cognitive processing abilities. Journal of Intelligence.

[B45-jintelligence-14-00262] Schneider W. J., Kaufman A. S. (2017). Let’s not do away with comprehensive cognitive assessments just yet. Archives of Clinical Neuropsychology.

[B46-jintelligence-14-00262] Schneider W. J., Reynolds C. R., McGrew K. S., Salekin K. L. (2026). Life-and-death psychometrics: Generalizable best methods for combining scores in intellectual disability and other diagnostic assessments. Journal of Pediatric Neuropsychology.

[B47-jintelligence-14-00262] Sternberg R. J., Detterman D. K. (1986). What is intelligence? Contemporary viewpoints on its nature and definition.

[B48-jintelligence-14-00262] Stuebing K. K., Fletcher J. M., LeDoux J. M., Lyon G. R., Shaywitz S. E., Shaywitz B. A. (2002). Validity of IQ-discrepancy classifications of reading disabilities: A meta-analysis. American Educational Research Journal.

[B49-jintelligence-14-00262] Tiekstra M., Minnaert A., Hessels M. G. P. (2016). A review scrutinising the consequential validity of dynamic assessment. Educational Psychology.

[B50-jintelligence-14-00262] Tough P. (2020). Go ahead, California, get rid of the SAT. The New York Times.

[B51-jintelligence-14-00262] Uttl B. (2005). Measurement of individual differences: Lessons from memory assessment in research and clinical practice. Psychological Science.

[B52-jintelligence-14-00262] van Hoogdalem A., Bosman A. M. T. (2024). Intelligence tests and the individual: Unsolvable problems with validity and reliability. Methodological Innovations.

[B53-jintelligence-14-00262] Warne R. T., Burningham C. (2019). Spearman’s *g* found in 31 non-Western nations: Strong evidence that *g* is a universal phenomenon. Psychological Bulletin.

[B54-jintelligence-14-00262] Westrick P. A., Angehr E. L., Shaw E. J., Marini J. P. (2024). Recent trends in college readiness and subsequent college performance: With faculty perspectives on student readiness.

[B55-jintelligence-14-00262] Worrell F. C. (2009). Myth 4: A single test score or indicator tells us all we need to know about giftedness. Gifted Child Quarterly.

